# Cross-cultural translation of the Western Ontario Cuff Index in Chinese and its validation in patients with rotator cuff disorders

**DOI:** 10.1186/s12891-017-1536-y

**Published:** 2017-05-02

**Authors:** Wei Wang, Qing-yun Xie, Zhen-yu Jia, Lin Cui, Da Liu, Cai-ru Wang, Wei Zheng

**Affiliations:** 1 Department of Orthopedics, Chengdu Military General Hospital, Tianhui Road 270, Chengdu, People’s Republic of China; 2 Department of Orthopedics, Changhai Hospital, Changhai Road 168, Shanghai, People’s Republic of China

**Keywords:** WORC, Scale, Validation, Cross-culture adaptation

## Abstract

**Background:**

The Western Ontario Rotator Cuff Index (WORC) is a scale designed to evaluate the impact of rotator cuff (RC) disorders on patients’ general quality of life. Our study aims to adapt the WORC for Chinese patients and to assess its reliability, validity, and responsiveness in Chinese patients with RC disorders.

**Methods:**

First, we developed the Chinese version of the WORC (C-WORC) in a five-step procedure of translation and cross-cultural adaptation. Next, the recruiting patients finished all three rounds of scales of the C-WORC, the Medical Outcomes Study Short-Form 36 (SF-36), and the Oxford Shoulder score (OSS). Then we calculated Cronbach’s alpha, the intra-class correlation coefficient (ICC), Pearson’s or Spearman’s correlation coefficient (*r* or *r*_s_), the effect size (ES), and the standardized response mean (SRM) to evaluate the reliability, validity and responsiveness of the C-WORC, respectively.

**Results:**

Overall, 124 patients with RC disorders successfully completed the first two rounds of the scales, and 108 patients completed the last round of the scales. Good or excellent internal consistency (Cronbach’s alpha = 0.872–0.954) was found in the overall scale and subscales of C-WORC, as well as good or excellent test-retest reliability (ICC = 0.828–0.961). Moderate or good correlations (*r/r*_s_ *=* 0.472–0.787) were obtained between the physical subscales of the C-WORC and the OSS and the physical subscales of SF-36; the results were also obtained for the emotions subscale of the C-WORC and the mental subscales of SF-36 (*r/r*_s_ *=* 0.520–0.713), which, adequately illustrated that good validity was included in the C-WORC. In addition, good responsiveness was also observed in the overall scale and subscales of the C-WORC (ES = 1.57–2.27, SRM = 1.52–2.28).

**Conclusions:**

The C-WORC scale is reliable, valid and responsible for the evaluation of Chinese-speaking patients with RC disorders and would be an effective instrument.

## Background

After back and neck pain, shoulder pain is the third most common musculoskeletal condition encountered in medical practice and causes significant disability [1]. Among shoulder pathologies, rotator cuff (RC) disorders are the most prevalent with 35–45% of rendered diagnoses [2]. RC disorders have negative impacts on the patient’s activities of daily living, work and sport activities, consequently influencing health-related quality of life (HRQOL) [3, 4].

A large body of research has been devoted to the development of the HRQOL scales since the 1980s [5]. The HRQOL scales are generally used to collect the relevant data through questionnaires completed independently by patients. Doctors can understand the severity of the patients’ condition by the information obtained through these scales and to develop a more appropriate treatment option for patients [6]. According to their applications, these scales can be classified as generic scales and disease-specific scales. The former are developed for the evaluation of the overall status of a patient, such as the commonly used Medical Outcomes Study Short-Form 36 (SF-36), while the latter may be applicable for specific patient populations, such as the Western Ontario Shoulder Instability Index (WOSI) for shoulder instability [7], the Western Ontario Osteoarthritis of the Shoulder Index (WOOS) for shoulder osteoarthritis [8], and the Rotator Cuff Quality of Life Index (RC-QOL) [9] and the Western Ontario Cuff Index (WORC) [10] for RC disorders.

Many scales are being used in different patient groups in different countries. This need has become more essential with the growing number of multicenter and multinational studies [5], which provide more statistical power of randomized controlled trials [11]. When one reliable, valid scale is being used in populations of different cultures, to avoid the evaluation error caused by cultural differences, it is necessary to test the psychometric properties of the scale rather than simply translating the content [12, 13].

Currently, only two scales that can be used in populations with shoulder disorders, the Disabilities of the Arm, Shoulder and Hand (DASH) and Oxford Shoulder score (OSS), have been translated, cross-culturally adapted and validated into Chinese [14, 15]. However, the DASH and OSS were specifically designed for patients with upper-extremity disorders and subacromial pain, respectively. Neither of these 2 scales is a disease-specific scale for Chinese-speaking patients with RC disorders.

The WORC is a newly developed self-administered disease-specific instrument that was designed to measure the HRQOL in patients with RC disorders [10]. The psychometric properties of the original WORC have been tested and have shown good reliability, validity and responsiveness [10, 16–18]. In a systematic review on the patient-reported outcomes used for the evaluation of symptoms and functional limitations in individuals with RC disorders, it was concluded that the WORC is one of the most responsive questionnaires for this population [19]. The original version of the WORC was created in English and has been translated and validated into 7 languages, including German, Dutch, Brazil, and Japanese, among others [20–26]. Unfortunately, a Chinese version has not yet been published even though China has the largest population of patients with RC disorders [27].

Therefore, we aimed to translate and adapt the WORC into a Chinese version (C-WORC) and evaluate the reliability, validity and responsiveness of the C-WORC in a cohort of native Chinese-speaking patients with RC disorders.

## Methods

### Translation and Cross-cultural Adaptation

The translation work of the WORC followed the principles of previously published guidelines [28, 29]. The entire process consisted of 5 steps: (1) Forward translation from English to Chinese by 2 bilingual translators independently (an orthopedic surgeon from our department and a professional translator). (2) For a synthesis of the translations, a discussion was held to integrate the 2 independent forward translation drafts; later we obtained the primary Chinese version of WORC (C-WORC). (3) Backward translation by 2 independent native English translators (OA and JR) who are well conversant in Chinese, the 2 translators have medical backgrounds, but no knowledge of the original WORC. (4) Creation of a pre-final version: a pre-final version was created by an expert committee after comparing the translated versions to the original version. (5) Twenty patients with RC disorders were invited to complete the pre-final C-WORC for assessment, and feedback was collected. A third meeting was conveyed by all research members for final adjustments according to this feedback, and the final version of the C-WORC was obtained.

### Patients and data collection

Patients enrolled in this study were mainly recruited from those with RC disorders admitted to Chengdu Military General Hospital and Changhai Hospital of Shanghai from January 2015 to March 2016. The inclusion criteria were patients > 18 years old with Chinese as their mother tongue; definitely diagnosed as RC by history, physical examination, and appropriate radiological evaluations and scheduled for arthroscopic shoulder surgery. The exclusion criteria were patients with chronic inflammatory diseases or impairments in the cervical spine, elbow, or hand affecting the shoulder function and/or with other systemic diseases. The number of patients enrolled also needed to meet the standard sample size of the health questionnaire proposed by Terwee et al. [30], that is, scale results from at least 100 patients for internal consistency analysis and at least 50 patients were required for the reliability and validity analysis. All patients who participated in the study had carefully read and signed informed consent, and the clinical study was approved by the ethics committee of our hospital (No. CHEC 2015–012).

The patients were asked to provide demographic information such as sex, age and weight on the first day of enrollment, and to independently complete the C-WORC, OSS, SF-36, and C-RC-QOL (for another study) in a quiet meeting room. One week after the first day of enrollment, also the day before the arthroscopic surgery, they completed the C-WORC for the second time to evaluate the test-retest reliability of the scale. Six months after the surgery, when the patients came to our hospital for a regular check, they completed the C-WORC for the third time to help evaluate the responsiveness.

### Scales

The WORC is a self-assessment scale that was developed to measure the quality of life of patients with RC disorders. It contains 21 items representing 5 subscales: physical symptoms (6 items), sports/recreation (4 items), work (4 items), lifestyle (4 items), and emotions (3 items), which encompass all aspects of heath as defined by the World Health Organization [31]. Each item is answered on a 100 - mm visual analog scale. The scores of 21 items are added to give a total score from 0 to 2100. To make scoring more understandable, the authors of the original version recommend that the data be converted to a percentage score by inverting the raw score and converting it to a score out of 100. A score of 0 is the worst score possible, and a score of 100 implies no reduction in the HRQOL [10]. If a response is lacking in any subscale, the lost item score can be compensated for by the mean of the other items in the subscale. Nevertheless, losses of more than two items in a subscale cannot be compensated, they must be listed as incomplete [32].

The OSS is also a self-assessment scale that was developed for patients with shoulder pain. It assesses the effect of shoulder joint disease on daily living and quality of life with 12 items [33]. Each item of the OSS has 5 answer options, each option corresponds to 0–4 points, and the score for the whole questionnaire ranges from 0 (worst) to 48 (best), indicating that patients with lower scores have worse shoulder joint function [34]. The SF-36 is a generic scale used to evaluate quality of life, and consists of 8 subscales with 36 items. Each subscale of the SF-36 has a special scoring method, and the final score is converted to a percentage (0–100). The lower the SF-36 score, the worse is the quality of life or functional status [35]. Chinese versions of these 2 scales are available that have been proven to have acceptable reliability, validity, and responsiveness [15, 36].

### Psychometric Assessments and Statistical Analysis

The reliability test of the C-WORC mainly included the evaluation of the test-retest reliability and internal consistency. The test-retest reliability of the scale was evaluated via the comparison of the first 2 rounds results’ of the C-WORC. The intraclass correlation coefficient (ICC), which was derived from a 2-way analysis of variance in a random effect model, was used as the evaluating index. The scale was considered to have good or excellent reliability when the ICC was > 0.8 or 0.9, respectively [37]. With Cronbach’s alpha as an evaluation index of the internal consistency of the C-WORC, the scale was deemed to show acceptable, good, or excellent internal consistency when this index was >0.7, 0.8, or 0.9, respectively [30]. We further depicted Bland-Altman plots to observe for systematic error between the first two rounds of investigations [38].

Then we assessed the validity of the C-WORC by evaluating both the content validity and construct validity. The content validity consists of the assessments of comprehensiveness and the relevance of items [39]. The item response rate, ceiling/floor effects, and patient feedback were the 3 indexes for comprehensiveness assessment. If the response rate for each item in the scale was >95%, the ceiling/floor effects of each subscale were <15%, and there were no difficulties in understanding the items that were fed back from the patients filling in the C-WORC, the scale was considered to have good comprehensiveness [30, 40]. In addition, a rehabilitation medicine expert and 3 orthopedics specialists were invited to help judge whether the items were relevant for the construct to be measured and for the population of patients with RC disorders [39]. Because there is no gold standard for evaluating the validity of the C-WORC, the hypotheses testing method was employed to evaluate the construct validity [39]. In this study, we selected the OSS and SF-36 as the control scales for the C-WORC. On the basis of the content of each scale, we hypothesized that the physical subscales of the C-WORC (physical symptoms, sports/recreation, work, life style) should be well correlated with the OSS and the physical subscales of SF-36 (physical functioning, role physical, bodily pain, general health), but poorly with the mental subscales of SF-36 (vitality, social functioning, role emotional, mental health). Correspondingly, the emotions subscale of the C-WORC should be well correlated with the mental subscales of SF-36, and poorly with the OSS and the physical subscales of SF-36. In addition, because the OSS is specifically designed to assess the patient’s function in the shoulder area, and the SF-36, however, is only a generic scale, we hypothesized that the correlation between the C-WORC and the OSS should be better than that of any subscales of SF-36. On the basis of the above hypotheses, we used the results derived from the first round investigation to calculate the correlation coefficient (*r* or *r*_s_) of the C-WORC with the subscales of SF-36 and the OSS. In addition, all the scores were tested for normal distribution using the Kolmogorov-Smirnov test, and Pearson’s (*r*) and Spearman’s (*r*_s_) correlation coefficients were used for parametric and non-parametric score data, respectively. The construct validity of the C-WORC was evaluated by comparing the compatibility of the results with our initial hypotheses. The correlations were judged as poor (*r/r*_s_ = 0–0.2), fair (*r/r*_s_ = 0.2–0.4), moderate (*r/r*_s_ = 0.4–0.6), good (*r/r*_s_ = 0.6–0.8), or excellent (*r* = 0.8–1.0) [41].

Finally, we evaluated the responsiveness of the C-WORC by comparing the scale results before and 6 months after arthroscopic surgery. Effect size (ES) and standardized response mean (SRM) were the 2 indices to evaluate the responsiveness. SRM was defined as the mean change between these time points divided by the SD of this change. The ES was defined as the mean change between the preoperative results and the 6-month postoperative results divided by the SD of the preoperative C-WORC score [42]. The ES and SRM were considered large if >0.80, moderate if between 0.51 and 0.80, and small if lower than 0.50 [43].

Statistical package for the Social Sciences, version 20.0 (SPSS, Chicago, IL, USA) was used for statistical analysis.

## Results

### Patients

Overall, 152 patients (74 male and 78 female) with RC disorders admitted to both of the hospitals from January 2015 to March 2016 met the inclusion/exclusion criteria, out of which 124 patients (81.6% of those invited,69 male and 55 female) finally agreed to participate in the study upon our invitation. All patients completed the scales for the first two rounds, and 16 patients did not visit the hospital again to complete the third round scale 6 months after shoulder arthroscopic surgery. Hence, the sample size for the reliability and validity assessment of the C-WORC was 124, while that for the responsiveness assessment was 108. The patients initially enrolled were an average age of 47.3 (ranging from 20–66), and the duration of pain was 30.7 months on average (ranging from 1–72 months). More detailed demographic information is summarized in Table 1.

**Table 1:** Demographic and clinical characteristics of participants

Characteristics	Number (%) or Mean ± SD
Age (years)	47.3 ± 9.5
Range	20 – 66
Age groups	
≦30	8 (6.5%)
31 – 45	40 (32.3%)
46 – 60	64 (51.6%)
≧61	12 (9.7%)
Gender	
Female	55 (44.4%)
male	69 (55.6%)
Affected side	
Right	64 (51.6%)
Left	60 (48.4%)
Dominant side	
Dominant	71 (57.3%)
Nondominant	53 (42.7%)
Pain duration (months)	30.7 ± 20.2
BMI (Kg/m^2^)	23.7 ± 4.6

*BMI* body mass index

### Translation and cross-culture adaptation process

The forward and backward translation of the WORC went smoothly. In the process of translation, only item 17 was slightly modified; “roughhousing or horsing around with family or friends” was changed to “roughhousing or horsing around with friends”, which is more suitable for the Chinese culture. Overall, 20 patients (10 male and 10 female) with RC disorders had completed the pre-final version of the C-WORC, and no participant complained of irregularities in the items or difficulties understanding the items. This version was used as the final version in the subsequent validation phase without any further change.

### Reliability

**Fig. 1:**
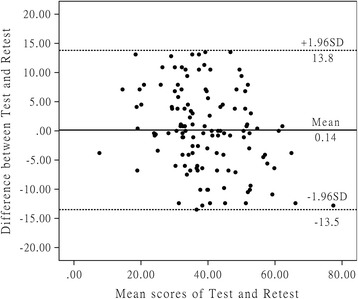
These are Bland-Altman plots of test-retest reliability of the C-WORC. Each data point indicates how the difference between the two test sessions for an individual patient compares to the mean of the two sessions for scores of each C-WORC. The interval of two sessions was 1 week. The dashed line shows the 95% (±1.96 SD) limits of agreement

The overall scale of the C-WORC had excellent internal consistency (Cronbach’s alpha = 0.950), and each subscale of the C-WORC showed good or excellent internal consistency (Cronbach’s alpha = 0.872–0.954) (Table 2). Moreover, the global test-retest reliability of the C-WORC was good (ICC = 0.893), and the test-retest reliability of each subscale was also good or excellent (ICC = 0.828–0.961) (Table 3). The Bland-Altman plots showed no systematic error between the results of the first two rounds (Fig. 1), which also confirmed and highlighted the good test-retest agreement of the C-WORC.

**Table 2:** Distribution and internal consistency for the subscales of the C-WORC

Subscale	Mean ± SD	Observed range	Theoretical range	Missing items n (%) ^a^	Floor effect (%) ^b^	Ceiling effect (%) ^b^	Cronbach's Alpha
Overall scale	37.1 ± 13.4	6.0 – 68.7	0 – 100	1 (0.8)	0	0	0.950
Physical symptoms	40.2 ± 15.1	0 – 82.5	0 – 100	0 (0)	1.6	0	0.938
Sports/recreation	33.2 ± 13.6	0 – 72.0	0 – 100	0 (0)	0.8	0	0.911
Work	30.9 ± 15.0	0.5 – 67.5	0 – 100	1 (0.8)	0	0	0.872
Lifestyle	38.1 ± 16.3	0 – 77.8	0 – 100	0 (0)	1.6	0	0.954
Emotions	42.9 ± 26.6	2.3 – 100	0 – 100	1 (0.8)	0	0.8	0.946

*C-WORC* Chinese version of the Western Ontario Rotator Cuff Index, *SD* Standard deviation

^a^Number of patients with some missing items in the subscale or overall scale

^b^Percentage of patients with the worst (floor effect) and the best (ceiling effect) score

**Table 3:** Construct validity, reliability, and responsiveness of the C-WORCC-WORC

Parameter	C-WORC subscale (No. items)					
	Physical symptom (6)	Sports/ Recreation (4)	Work (4)	Lifestyle (4)	Emotions (3)	Overall scale (21)
*Construct validity indicated by correlation coefficient r r*_*s*_ *(P value) with indicated instruments* ^a,b^						
OSS	0.774 (<0.001)	0.787 (<0.001)	0.732 (<0.001)	0.672 (<0.001)	0.242 (0.007)	0.842 (<0.001)
SF-36						
Physical function	0.523 (<0.001)	0.580 (<0.001)	0.532 (<0.001)	0.472 (<0.001)	0.242 (0.007)	0.568 (<0.001)
Role physical	0.514 (<0.001)	0.598 (<0.001)	0.622 (<0.001)	0.532 (<0.001)	0.144 (0.111)	0.605 (<0.001)
Bodily pain	0.623 (<0.001)	0.655 (<0.001)	0.621 (<0.001)	0.594 (<0.001)	0.289 (0.001)	0.663 (<0.001)
General health	0.540 (<0.001)	0.497 (<0.001)	0.510 (<0.001)	0.485 (<0.001)	0.126 (0.165)	0.579 (<0.001)
Vitality	0.180 (0.045)	0.219 (0.015)	0.183 (0.042)	0.181 (0.045)	0.583 (<0.001)	0.248 (0.006)
Social function	0.286 (0.001)	0.309 (<0.001)	0.350 (<0.001)	0.222 (0.013)	0.520 (<0.001)	0.326 (0.001)
Role emotional	0.350 (<0.001)	0.368 (<0.001)	0.226 (0.011)	0.275 (0.002)	0.713 (<0.001)	0.391 (<0.001)
Mental health	0.321 (<0.001)	0.342 (<0.001)	0.352 (<0.001)	0.261 (0.003)	0.571 (<0.001)	0.366 (<0.001)
*Test-retest reliability, mean (SD) or ICC value (CI range)* ^b^						
Test score	40.2 (15.1)	33.2 (13.6)	30.9 (15.0)	38.1 (16.3)	42.9 (26.6)	37.1 (13.4)
Retest score	40.1 (16.3)	33.1 (15.7)	30.7 (17.3)	38.0 (17.4)	42.7 (27.0)	36.9 (15.0)
Score change	−0.1 (5.9)	−0.1 (7.1)	−0.2 (9.5)	−0.1 (4.7)	−0.2 (8.8)	−0.1 (6.6)
ICC (95% CI)	0.930 (0.902–0.951)	0.885 (0.840–0.918)	0.828 (0.763–0.866)	0.961 (0.945–0.973)	0.946 (0.924–0.962)	0.893 (0.851–0.924)
*Responsiveness pre-treatment vs 6 months after arthroscopic treatment, mean (SD)* ^c^						
Pre-treatment score	40.5 (14.5)	33.4 (13.0)	31.2 (14.5)	38.4 (16.1)	42.9 (26.5)	37.3 (16.2)
Post-treatment score	73.5 (19.4)	62.3 (17.2)	53.9 (21.4)	69.5 (19.0)	85.1 (17.1)	68.5 (19.0)
Score change	33.0 (16.6)	28.9 (15.1)	22.8 (17.9)	31.2 (18.6)	42.2 (27.6)	31.2 (13.7)
ES	2.27	2.22	1.57	1.94	1.59	1.92
SRM	1.99	1.91	1.27	1.68	1.52	2.28

*C-WORC* Chinese version of the Western Ontario Rotator Cuff Index, *OSS* Shoulder Oxford score, *SD* Standard deviation, *ICC* Intraclass correlation coefficient, *CI* Confidence interval, *ES* Effect size, *SRM* Standardized response mean

^a^Calculated by the Pearson correlation coefficient (*r*) or Spearman’s correlation coefficient (*r*s) of the C-WORC with OSS and SF-36

^b^The sample size for the analysis of construct validity and test-retest reliability was 124

^c^The sample size for the analysis of responsiveness was 108

### Validity

In the formal study, there was one item not answered in both the work and emotions subscales (1/124, 0.8%), and the 2 items missed appeared in the same questionnaire (Table 2). The average score of the subscale and each subscale was 30.9–42.9, and the emotions subscale had the highest average score and the work subscale had the lowest score (Table 2). The overall scale and each subscale of the C-WORC did not show a ceiling effect (0–0.8%) and a floor effect (0–1.6%). In addition, after the completion of the C-WORC, no patients reported any difficulties in understanding any items in the scale. Content analysis of the C-WORC was performed by the rehabilitation medical expert and orthopedics specialists and it was agreed that the data derived from each individual item in the C-WORC were sufficient for the HRQOL evaluation of patients with RC disorders. Therefore, no addition or deletion of any items was recommended. On the basis of the above results, we believed that the C-WORC had good content validity.

Data for construct validity assessment were listed in Table 3. The scores of the Sports/Recreation subscale (C-WORC) and all subscales of SF-36 (excluding the GH subscale) were not normally distributed, so Spearman’s (*r*_s_) correlation coefficient was used for these subscales. The correlations between the physical subscales of the C-WORC and the OSS and the physical subscales of SF-36 were at least moderate (*r/r*_s_ = 0.472–0.787; *P* < 0.001), and that with the mental subscales of SF-36 were poor or fair (*r/r*_s_ = 0.180–0.368; *P* = <0.001–0.045). At the meanwhile, the correlations between the emotions subscale of the C-WORC and the mental subscales of SF-36 were at least moderate (*r/r*_s_ = 0.520–0.713; *P* < 0.001), and with the physical subscales of SF-36 and the OSS were poor or fair (*r/r*_s_ = 0.126–0.289; *P* = 0.001–0.045). In addition, the correlations between the physical subscales of the C-WORC and the OSS (*r/r*_s_ = 0.672–0.787; *P* < 0.001) were stronger than that with the physical subscales of SF-36 (*r/r*_s_ = 0.472–0.655; *P* < 0.001). The above results were consistent with our hypothesis, so it could be interpreted that the C-WORC has good construct validity.

### Responsiveness

Finally, we evaluated the responsiveness of the C-WORC by comparing the scales completed before and after arthroscopic surgery. Relevant data were listed in Table 3. In general, the average scores of the overall scale and other subscales had all increased after the arthroscopic surgery. Both the ES (1.57–2.27) and SRM (1.52–2.28) values exceeded 1.00, suggesting good responsiveness to the C-WORC.

## Discussion

The HRQOL scale is an important instrument in clinical studies. Researchers can quantify the functional status of patients and also compare these data with that derived from other scales. Clinical research is now developing rapidly in China, with a large number of relevant articles published every year. This can be explained by both the largest number of patient populations in China and the attention of the government to the scientific research [44]. Currently, effective scale instruments are needed in China to support the enormous clinical studies. Thus far, there are no disease-specific scales available in China that can be used to evaluate patients with RC disorders, a common problem that imposes a considerable burden on the affected person and society [3, 4]. The WORC, however, is currently the most widely used scale for the functional status evaluation of patients with RC disorders. It has been translated into 7 versions in different languages, and is proved to have acceptable reliability, validity and responsiveness [10, 16–20]. Therefore, we believe that it is of great importance to translate and adapt the WORC into Chinese, a language used by the largest number of people in the world, and that is the main objective of our study.

Prior to the discussion of the study results, it is important to note the limitations of this study. First, the sample was limited in size and may not fully represent the Chinese population. Second, the target language we want to translate for is the simplified Chinese, which is the official language in China. However, China is a multi-ethnic country, with many ethnic minorities with their own languages. Therefore, attention must be paid to national cultural differences when the C-WORC is employed. Finally, no effect was assessed in the C-WORC for the patients with RC disorders who had received conservative treatment, and this should be carried out in follow-up studies.

In this study, the process of translation and cross-cultural adaptation has been conducted smoothly, and we only slightly modified the content of item 17. Because generations of people live together in most traditional Chinese families and people are rarely “roughhousing or horsing around” with their own family members, especially with the elders in the family, so we have made corresponding changes to the subject in order to adapt to the Chinese culture. In the preliminary analysis and the formal research process, no incomprehensible items in the C-WORC were fed back from the patients.

The overall scale of the C-WORC and all the subscales had good or excellent internal consistency, which was consistent with other cross-culture adaptation studies and the original version (Cronbach’s alpha = 0.78–0.98) [10, 20–26]. The overall scale of the C-WORC and all the subscales also showed good or excellent test-retest reliability. The lifestyle subscale had the highest ICC value, which might be possibly explained by the constant daily living routine within 1 week. In addition, we believed that it is appropriate to choose 1 week as the interval time for the test-retest reliability assessment, because 1 week is long enough to allow patients to forget the specific answers they offered in the last questionnaires filing, while their functional status and life style remain unchanged within 1 week, and 1 week is exactly the time waiting for the arthroscopic surgery, during which no other treatments are generally administered to patients to avoid any relevant errors.

No ceiling or floor effect was observed in the overall scale of the C-WORC and all the subscales. Expert assessment also confirmed that the C-WORC’s items are good relevant for the construct to be measured and for the RC patient population. Although there was one item not answered in both the work and emotions subscales, it was the same patient who missed answering it. Therefore, we believed that the situation was more likely caused by personal factors, rather than the reasons for the scale itself. Integrating these results, we considered that the C-WORC has good content validity.

Correlations between the C-WORC and the subscales of SF-36 and the OSS were generally consistent with our hypotheses, suggesting that it has good construct validity, and these results also were in accordance with relevant conclusions from other studies [20–23, 25, 26]. The correlation between the C-WORC and the OSS is the strongest, despite the fact that the OSS is not specifically developed for patients with RC disorders. But the OSS has focused on the status of shoulder function and symptoms, just as the WORC does. Although the physical subscales of SF-36 were strongly associated with the C-WORC, it was still lower than that between the C-WORC and the OSS. This is because that the accuracy of SF-36, as a generic scale, in the functional status assessment of specific types of patients is lower than that of other specific scales [45]. Furthermore, correlations between the mental subscales and physical subscales of SF-36 and the C-WORC were poor, and this result was logical and consistent with that of other studies [21–23, 25, 26].

The responsiveness of a scale is an important factor to determine whether it can be used in a prospective clinical study. The results of our study showed that the overall scale of the C-WORC and its subscales have good responsiveness, suggesting that it can sensitively detect the changes in the functional status of patients who underwent arthroscopic surgery. ES and SRM values in our study, however, were slightly greater than other relevant studies (ES = 0.96–1.35, SRM = 0.91–1.54) [20, 24, 26]. This is possibly explained by the fact that the treatment our patients received was arthroscopic surgery, and surgical operation as well as conservative treatment was included in other studies, resulting in the different improvement in functional status.

## Conclusions

In summary, we successfully translated and adapted the WORC into a Chinese version, which was proven to have good reliability, validity, and responsiveness. We therefore suggest that the C-WORC can be used in the functional status evaluation of patients with RC disorders in future clinical studies performed in Chinese populations, so as to help doctors or researchers collect data needed.

## Abbreviations

C-RC-QOL: Chinese version of rotator cuff quality of life index
DASH: Disability of arm shoulder and hand
ES: Effect size
HRQOL: Health-related quality of life
ICC: Intra-class correlation coefficient
OSS: Oxford shoulder score
RC: Rotator cuff
RC-QOL: Rotator cuff quality of life index
SF-36: Short-Form 36
SRM: Standardized response mean
WOOS: Western Ontario osteoarthritis of the shoulder index
WORC: Western Ontario Cuff Index
WOSI: Western Ontario shoulder instability index
